# Long‐Term Postoperative Donor Site Musculoskeletal Morbidity after Osseous Free Flap Harvest

**DOI:** 10.1002/oto2.70069

**Published:** 2025-01-10

**Authors:** Tyler G. Chan, Aaron Rosado, Subir Goyal, Rachel Irizarry, Robert J. Owen, Harry Michael Baddour, Brian Boyce, Azeem Kaka, Mark W. El‐Deiry, Jennifer H. Gross

**Affiliations:** ^1^ Emory University School of Medicine Atlanta Georgia USA; ^2^ Biostatistics Shared Resource Winship Cancer Institute Emory University Atlanta Georgia USA; ^3^ Department of Otolaryngology–Head and Neck Surgery, Winship Cancer Institute Emory University Atlanta Georgia USA; ^4^ Peachtree Orthopedics Atlanta Georgia USA

**Keywords:** donor site, functional status, musculoskeletal morbidity, osseous free flap harvest, quality of life

## Abstract

**Objective:**

Complex ablative maxillary and mandibular defects often require osseous free flap reconstruction. Workhorse options include the fibula, scapula, and osteocutaneous radial forearm flap (OCRFF). The choice of donor site for harvest should be driven not only by reconstructive goals but also by donor site morbidity. The goal of this study is to evaluate the long‐term postoperative musculoskeletal morbidity at the donor site after osseous free flap harvest.

**Study Design:**

Cohort study and cross‐sectional analysis.

**Methods:**

A retrospective review of patients who underwent free flap harvest at 1 of the 3 donor sites from 2015 through 2021 was performed. An additional cross‐sectional analysis at ≥1 year postoperatively was performed from 2021 to 2022 using validated patient‐reported orthopedic surveys: Disabilities of the Arm, Shoulder, and Hand for scapula or OCRFF harvest, and Foot and Ankle Ability Measure for fibula harvest.

**Setting:**

Single, high‐volume tertiary care institution.

**Results:**

Among 731 eligible patients, 162 (22.1%) answered the telephone surveys and were included. Functional differences between operated and nonoperated sides were 18.5% (scapula, n = 33), 13.5% (OCRFF, n = 29), and 10% (fibula, n = 98). Postoperative physical therapy (for all donor sites), ipsilateral neck dissection (for scapula and OCRFF), and extent of bony resection (for OCRFF) were not factors associated with long‐term morbidity. Acute donor site complications were most common in fibula patients and were associated with worse long‐term functional outcomes (7.5% difference; 95% confidence interval, −14.0 to −1; *P* = .03).

**Conclusion:**

There is acceptable long‐term musculoskeletal morbidity at the donor site after osseous free flap harvest, and patients should be counseled appropriately.

Microvascular free flaps are essential to the contemporary reconstruction of head and neck defects. When reconstruction of the mandible or midface requires bone, osseous free flaps have become standard‐of‐care to reconstruct acquired defects.^1^, ^2^ Many donor sites now exist with the optimal choice dictated chiefly by requirements of the defect, functional goals of the reconstruction, and patient factors.^3^, ^4^ Historically, the first report of an osseous free flap for head and neck reconstruction was in 1977 by Serafin et al who harvested a rib‐containing free flap to reconstruct a mandibular defect.^5^ Today, 3 donor sites in particular account for the majority of osseous‐free flaps performed: the scapula‐free flap (SFF), osteocutaneous radial forearm flap (OCRFF), and fibula‐free flap (FFF).^6^ Each has its benefits and drawbacks.^7^ Given their low rate of postoperative complications, high level of patient satisfaction with recipient site functional and aesthetic results, and favorable survival outcomes, attention has now turned to donor site functional morbidity.^8^, ^9^

While recipient site outcomes are favorable, the degree of long‐term postoperative musculoskeletal disability following SFF, OCRFF, and FFF remains unclear.^10^, ^11^, ^12^, ^13^, ^14^, ^15^, ^16^, ^17^, ^18^, ^19^ Many studies have explored this issue; however, few have used patient‐reported outcome measures.^20^, ^21^, ^22^, ^23^, ^24^, ^25^, ^26^, ^27^, ^28^, ^29^, ^30^, ^31^ Furthermore, none to our knowledge compare functional morbidity across all 3 donor sites above. Proper understanding of subjective reports of functional status and postoperative quality of life is critical for preoperative counseling regarding the optimal donor site for each patient and therefore warrants further investigation.

The primary goal of this study is to use a database and the results from validated orthopedic surveys to review the degree of long‐term postoperative donor site musculoskeletal disability after SFF, OCRFF, and FFF harvest. Based on evidence from previous reports, we hypothesize there to be a minimal to mild difference in functional morbidity between operated and nonoperated sides.^21^, ^23^ Secondary objectives include determining risk factors impacting the morbidity of each donor site, type of postoperative complications, and rates of dental rehabilitation and physical therapy (PT) for each donor site.

## Methods

This cohort study and cross‐sectional analysis were approved by the Institutional Review Board at Emory University, including approval of the telephone‐administered surveys. A retrospective cohort study was performed on patients ≥18 years old who underwent osseous free flap harvest (SFF, OCRFF, and FFF) for head and neck reconstruction at a single, high‐volume institution (Emory University Hospital Midtown) from January 2015 to December 2021. All eligible patients were identified through a departmental billing database using CPT codes 20969 (free osteocutaneous flap with microvascular anastomosis) and 20955 (fibula bone graft with microvascular anastomosis). Patients who had a prior free flap performed in the same or adjacent recipient or donor site were excluded. Collected and analyzed data included demographics, insurance coverage, pathologic diagnosis, operative details, multimodal treatment details, recipient and donor site postoperative complications, percutaneous endoscopic gastrostomy (PEG) tube dependence, participation in postoperative PT, and dental rehabilitation (implants or dentures). Operative details included defect type, length of bone harvested (for OCRFF), location of bony harvest ± latissimus (for SFF), closure of donor site (primary vs requiring split‐thickness skin graft [STSG]), and presence of ipsilateral neck dissection (for SFF and OCRFF). Data were acquired by review of Emory Healthcare electronic health records.

An additional cross‐sectional analysis at ≥1 year postoperatively was performed from 2021 to 2022 using validated patient‐reported orthopedic surveys as primary outcome measures. The 2 surveys employed were Disabilities of the Arm, Shoulder, and Hand (DASH)^32^ for SFF and OCRFF, and the Foot and Ankle Ability Measure (FAAM)^33^ score for FFF. The DASH score measures disability and symptoms in patients with upper extremity disorders, with 30 questions addressing physical function, pain, and emotional/social function. Scores range from 0 (no disability) to 100 (severe disability). The FAAM score assesses disability in patients with disorders of the foot and ankle, using 21 questions that address activities of daily living. Scores range from 0% (severe disability) to 100% (no disability). Surveys were completed over the phone after verbal consent was obtained. Scores from these surveys were calculated and used to evaluate long‐term postoperative donor site musculoskeletal function. The amount of time elapsed between the date of surgery and the date of survey completion was ≥1 year, but different in each patient. This time period was chosen based upon previous literature showing a precipitous decrease in functional morbidity up until 1 year postoperatively, after which functional morbidity tends to decrease symmetrically between both limbs when controlling for aging.^34^, ^35^ The appropriate survey was administered over the telephone for both the surgical site and the contralateral limb as an internal control.

Statistical analysis was conducted using SAS statistical software version 9.4 (SAS Institute Inc). Demographic and clinical characteristics were summarized using descriptive statistics. We performed univariate analyses to estimate the differences in the postoperative musculoskeletal function between the surgical side and contralateral side for all 3 donor sites by using paired *t* tests. Multivariable analyses were performed by using mixed models to determine intraoperative and perioperative factors impacting either DASH or FAAM scores. The significance level (*α*) was set at .05. We calculated confidence intervals (CIs) at a confidence level of 95%, and hypothesis tests were 2‐sided.

## Results

### Demographics and Surgical Factors

Between January 2015 and December 2021, 731 osseous free flaps were performed at our institution by 7 surgeons. In total, 162 patients (22.1%) answered either DASH or FAAM surveys and were therefore included in the study. Among the 33 patients who underwent SFF (median [SDV] age, 64 [10.2] years; 20 [60.6%] men), 1 patient (3.0%) underwent a scapula tip, 22 (66.7%) underwent a scapular border, and 10 (30.3%) underwent a chimeric scapular border with the latissimus dorsi muscle (Table 1). The scapula tip patient required the shortest amount of bone at 8 cm. Among 29 patients who underwent OCRFF (median [SDV] age, 63 [12.46] years; 18 [62.1%] men), the average length of radial bone taken was 6.5 cm [1.55] (Table 2). Finally, 98 patients underwent a fibula flap (median [SDV] age, 59.5 [14.8] years; 53 [54.1%] men) (Table 3).

**Table 1:** Scapula Flap Patient Demographics and Perioperative Characteristics

Variable		N (%) = 33
Gender	Male	20 (60.6)
	Female	13 (39.4)
Insurance	None	1 (3.0)
	Private	13 (39.4)
	Medicare	16 (48.5)
	Medicaid	2 (6.1)
	Government	1 (3.0)
Pathologic diagnosis	SCCa	18 (54.5)
	Verrucous carcinoma	2 (6.1)
	Adenoid cystic	3 (9.0)
	Benign tumor	1 (3.0)
	Neuroendocrine tumor	1 (3.0)
	ORN	2 (6.1)
	Mucoepidermoid carcinoma	1 (3.0)
	Odontogenic keratocyst	2 (6.1)
	Osteosarcoma	1 (3.0)
	Osteomyelitis	1 (3.0)
	Ameloblastoma	1 (3.0)
Stage	None	6 (18.2)
	I	1 (3.0)
	II	2 (6.1)
	III	3 (9.1)
	IVa	12 (36.4)
	IVb	8 (24.2)
	Not identifiable	1 (3.0)
Defect	Midface	8 (25.0)
	Mandible	23 (71.9)
	Chest	1 (3.0)
	Midface and mandible	1 (3.0)
STSG to the donor site	No	31 (93.9)
	Yes	2 (6.1)
Donor site complication	No	33 (100.0)
Flap recipient site complication	No	19 (57.6)
	Yes	14 (42.4)
Flap site complication type	None	19 (57.6)
	Seroma	2 (6.1)
	Infection	2 (6.1)
	Wound dehiscence	3 (9.1)
	Fistula	3 (9.1)
	Hardware exposure/failure	2 (6.1)
	>1 Complication	2 (6.1)
PEG dependence at 1 year	No	23 (69.7)
	Yes	10 (30.3)
Radiation	None	10 (30.3)
	Before surgery	8 (24.2)
	After surgery	15 (45.5)
Chemotherapy	None	17 (51.5)
	Before surgery	8 (24.2)
	After surgery	8 (24.2)
Immunotherapy	None	32 (97.0)
	After surgery	1 (3.0)
Dental rehabilitation	None	32 (97.0)
	Implant	1 (3.0)
Postoperative PT	No	12 (36.4)
	Yes	21 (63.6)
Scapula site	Scapula tip	1 (3.0)
	Scapular border	22 (66.7)
	Chimeric with latissimus dorsi	10 (30.3)
Ipsilateral neck dissection	No	13 (39.4)
	Yes	20 (60.6)
Age	Mean	63.3
	Median	64.0
	Minimum	29.0
	Maximum	82.0
	Standard deviation	10.2
DASH score ipsilateral	Mean	24.6
	Median	19.8
	Minimum	0.0
	Maximum	85.0
	Standard deviation	22.4
DASH score contralateral	Mean	6.1
	Median	0.0
	Minimum	0.0
	Maximum	77.6
	Standard deviation	16.6

Abbreviations: DASH, Disabilities of the Arm, Shoulder, and Hand; ORN, osteoradionecrosis; PEG, percutaneous endoscopic gastrostomy; PT, physical therapy; SCCa, squamous cell carcinoma antigen; STSG, split‐thickness skin graft.

**Table 2:** OCRFF Patient Demographics and Perioperative Characteristics

Variable		N (%) = 29
Gender	Male	18 (62.1)
	Female	11 (37.9)
Insurance	None	4 (13.8)
	Private	9 (31.0)
	Medicare	15 (51.7)
	Medicaid	1 (3.4)
Pathologic diagnosis	SCCa	21 (72.4)
	Hardware infection or bone fracture	1 (3.4)
	Adenocarcinoma	1 (3.4)
	Basal cell carcinoma	1 (3.4)
	ORN	2 (6.9)
	Mucoepidermoid carcinoma	1 (3.4)
	Chronic inflammation	1 (3.4)
	Fibrous dysplasia	1 (3.4)
Stage	None	6 (20.7)
	I	1 (3.4)
	II	4 (13.8)
	III	2 (6.9)
	IVa	12 (41.4)
	IVb	4 (13.8)
Defect	Midface	9 (31.0)
	Mandible	20 (69.0)
STSG to the donor site	No	2 (6.9)
	Yes	27 (93.1)
Donor site complication	No	28 (96.6)
	Yes	1 (3.4)
Flap recipient site complication	No	20 (69.0)
	Yes	9 (31.0)
Flap site complication type	None	20 (69.0)
	Fistula	4 (13.8)
	Hardware exposure/failure	2 (6.9)
	>1 Complication	3 (10.3)
PEG dependence at 1 year	No	24 (82.8)
	Yes	5 (17.2)
Radiation	None	15 (51.7)
	Before surgery	3 (10.3)
	After surgery	10 (34.5)
	Both	1 (3.4)
Chemotherapy	None	22 (75.9)
	Before surgery	2 (6.9)
	After surgery	5 (17.2)
Immunotherapy	None	25 (86.2)
	Before surgery	1 (3.4)
	After surgery	3 (10.3)
Dental rehabilitation	None	27 (93.1)
	Denture	2 (6.9)
Postoperative PT	No	18 (62.1)
	Yes	11 (37.9)
Ipsilateral neck dissection	No	12 (41.4)
	Yes	17 (58.6)
Age	Mean	61.7
	Median	63.0
	Minimum	26.0
	Maximum	78.0
	Standard deviation	12.5
Length of radial bone taken, cm	Mean	6.5
	Median	6.0
	Minimum	4.0
	Maximum	10.0
	Standard deviation	1.6
DASH score ipsilateral	Mean	15.6
	Median	9.8
	Minimum	0.0
	Maximum	50.9
	Standard deviation	15.3
DASH score contralateral	Mean	2.1
	Median	0.0
	Minimum	0.0
	Maximum	26.8
	Standard deviation	5.2

Abbreviations: DASH, Disabilities of the Arm, Shoulder, and Hand; OCRFF, osteocutaneous radial forearm flap; ORN, osteoradionecrosis; PEG, percutaneous endoscopic gastrostomy; PT, physical therapy; SCCa, squamous cell carcinoma antigen; STSG, split‐thickness skin graft.

**Table 3:** Fibula Flap Patient Demographics and Perioperative Characteristics

Variable		N (%) = 98
Gender	Male	53 (54.1)
	Female	45 (45.9)
Insurance	None	3 (3.1)
	Private	53 (54.1)
	Medicare	37 (37.8)
	Medicaid	5 (5.1)
Pathologic diagnosis	SCCa	41 (41.8)
	Adenoid cystic	4 (4.1)
	Benign tumor	2 (2.0)
	Osteomyelitis	5 (5.1)
	Hardware infection or bone fracture	3 (3.1)
	Adenocarcinoma	1 (1.0)
	Basal cell carcinoma	2 (2.0)
	ORN	16 (16.3)
	Odontogenic keratocyst	2 (2.0)
	Ameloblastoma	15 (15.3)
	Chronic inflammation	2 (2.0)
	Benign cemento osseous dysplasia	1 (1.0)
	Osteosarcoma	2 (2.0)
	Odontogenic carcinoma	1 (1.0)
	MRONJ	1 (1.0)
Stage	None	48 (49.0)
	I	1 (1.0)
	II	9 (9.2)
	III	5 (5.1)
	IVa	28 (28.6)
	IVb	7 (7.1)
Defect	Midface	11 (11.2)
	Mandible	87 (88.8)
STSG to the donor site	No	38 (38.8)
	Yes	60 (61.2)
Donor site complication	No	73 (74.5)
	Yes	25 (25.5)
Flap recipient site complication	No	68 (69.4)
	Yes	30 (30.6)
Flap site complication type	None	66 (67.3)
	Hematoma	1 (1.0)
	Infection	2 (2.0)
	Wound dehiscence	10 (10.2)
	Fistula	2 (2.0)
	Flap failure	2 (2.0)
	Hardware exposure/failure	4 (4.1)
	Sialocele	1 (1.0)
	Necrosis/debridement	1 (1.0)
	>1 Complication	9 (9.2)
PEG dependence at 1 year	No	82 (83.7)
	Yes	16 (16.3)
Radiation	None	37 (37.8)
	Before surgery	18 (18.4)
	After surgery	39 (39.8)
	Both	4 (4.1)
Chemotherapy	None	70 (71.4)
	Before surgery	16 (16.3)
	After surgery	11 (11.2)
	Both	1 (1.0)
Immunotherapy	None	91 (92.9)
	Before surgery	3 (3.1)
	After surgery	4 (4.1)
Dental rehabilitation	None	80 (82.5)
	Denture	4 (4.1)
	Implant	13 (13.4)
	Both	1 (1.0)
Postoperative PT	No	72 (73.5)
	Yes	26 (26.5)
Age	Mean	57.3
	Median	59.5
	Minimum	15.0
	Maximum	85.0
	Standard deviation	14.8
FAAM score ipsilateral	Mean	86.0%
	Median	95.0%
	Minimum	15.0%
	Maximum	100%
	Standard deviation	20.0%
FAAM score contralateral	Mean	96.0%
	Median	100%
	Minimum	21.0%
	Maximum	100%
	Standard deviation	13.0%

Abbreviations: FAAM, Foot and Ankle Ability Measure; MRONJ, medication‐related osteonecrosis of the jaw; ORN, osteoradionecrosis; PEG, percutaneous endoscopic gastrostomy; PT, physical therapy; SCCa, squamous cell carcinoma antigen; STSG, split‐thickness skin graft.

### Postoperative Outcomes

Among 33 scapula patients, PT was performed in 21 (63.6%) patients, 10 were PEG dependent at 1 year (30.3%), and no patients had a donor site complication (Table 1). Fourteen patients (42%) did have a recipient site wound complication. One patient (3%) ultimately had dental rehabilitation with implants.

Among 29 OCRFF patients, PT was performed in 11 (39.3%), 5 were PEG dependent at 1 year (17.6%), 1 (3.6%) experienced donor site complications, and 2 (7.1%) underwent dental rehabilitation with dentures (Table 2). Thirty‐two percent of patients (9/29) had a recipient site complication.

Among 98 fibula flap patients, PT was performed in 26 (26.5%), 16 were PEG dependent at 1 year (16.3%), 25 (25.5%) experienced donor site complications, and 17 (17.5%) underwent dental rehabilitation with dentures or implants (Table 3). Thirty percent of patients (30/98) had a recipient site complication, the most frequent of which was wound dehiscence (10/30).

### DASH and FAAM Scores With Multivariable Analysis

Mean ipsilateral versus contralateral DASH scores after scapula flap were 24.6 versus 6.1 (18.5% difference; 95% CI, 12.9‐24.2; *P* < .0001) (Table 4). On multivariable analysis, neither ipsilateral neck dissection during surgery (1.1% difference; 95% CI, −12.8 to 14.7; *P* = .9), nor postoperative PT (10.5% difference; 95% CI, −3.0 to 24.4; *P* = .1) was associated with a change in DASH score. Harvesting a chimeric latissimus muscle, when compared to the scapular border alone, was not associated with a change in DASH score (0.8% difference; 95% CI, −13.8 to 15.4; *P* = .9). The 1 scapula tip patient was combined with the scapular border patients for analysis because the harvest technique (which included transection of the teres major muscle) was similar.

**Table 4:** Multivariable Analysis of Factors Impacting DASH Score in Postoperative Scapula Patients

Factors	Test vs control	DASH score difference, % (95% CI)	*P* value
Group	Ipsilateral	18.5 (12.9‐24.2)	<.0001
	Contralateral	‐	
Postoperative PT	Yes	10.5 (−3.0 to 24.0)	.1
	No	‐	
Ipsilateral neck dissection	Yes	1.1 (−12.8 to 14.7)	.9
	No	‐	
Scapula site	Chimeric with latissimus dorsi	0.8 (−13.8 to 15.4)	.9
	Scapula tip + border	‐	

Abbreviations: CI, confidence interval; DASH, Disabilities of the Arm, Shoulder, and Hand; PT, physical therapy.

Mean ipsilateral versus contralateral DASH scores after OCRFF were 15.6 versus 2.1 (13.5% difference; 95% CI, 8.1‐19.5; *P* < .0001) (Table 5). On multivariable analysis, neither ipsilateral neck dissection during surgery (3.4% difference; 95% CI, −10.3 to 3.5; *P* = 0.3), nor each additional cm of harvested radius (0.6% difference per cm; 95% CI, −1.6 to 2.8; *P* = .6), nor postoperative therapy (5.9% difference; 95% CI, −1.0 to 12.8; *P* = .09) was associated with a change in DASH score.

**Table 5:** Multivariable Analysis of Factors Impacting DASH Score in Postoperative OCRFF Patients

Factors	Test vs control	DASH score difference, % (95% CI)	*P* value
Group	Ipsilateral	13.5 (8.1‐19.5)	<.0001
	Contralateral	‐	
Postoperative PT	Yes	5.9 (−1.0 to 12.8)	.09
	No	‐	
Ipsilateral neck dissection	Yes	3.4 (−10.3 to 3.5)	.3
	No	‐	
Length of radial bone taken, cm		0.6 (−1.6 to 2.8)	.6

Abbreviations: CI, confidence interval; DASH, Disabilities of the Arm, Shoulder, and Hand; OCRFF, osteocutaneous radial forearm flap; PT, physical therapy.

The mean ipsilateral versus contralateral FAAM score after a fibula flap was 86.0% versus 96.0% for the nonoperated side (10.0% difference; 95% CI, −14.0 to −7.0; *P* < .0001) (Table 6). On multivariable analysis, neither receiving an STSG to the donor site (4.1% difference; 95% CI, −2.0 to 10.0; *P* = .2) nor postoperative PT therapy (3.2% difference; 95% CI, −9.0 to 4.0; *P* = .4) was associated with a change in FAAM score. Patients with an acute donor site wound complication did have worse long‐term FAAM scores (7.5% difference; 95% CI, −14.0 to −1.0; *P* = .03).

**Table 6:** Multivariable Analysis of Factors Impacting FAAM Score in Postoperative Fibula Flap Patients

Factors	Test vs control	FAAM score difference, % (95% CI)	*P* value
Group	Ipsilateral	10.0 (−14.0 to −7.0)	<.0001
	Contralateral	‐	‐
Postoperative PT	Yes	3.2 (−9.0 to 4.0)	.4
	No	‐	‐
STSG to the donor site	Yes	4.1 (−2.0 to 10.0)	.2
	No	‐	‐
Donor site complication	Yes	7.5 (−14.0 to −1.0)	.03
	No	‐	‐

Abbreviations: CI, confidence interval; FAAM, Foot and Ankle Ability Measure; PT, physical therapy; STSG, split‐thickness skin graft.

## Discussion

### Scapula Flaps

When compared to OCRFF, our study shows that patients undergoing scapula flap reconstruction had the greatest difference (18.5%) in function between the operated and nonoperated side after at least 1 year, with a postoperative mean (SD) DASH score of 24.6 (22.4) (Table 1). A score of 21 to 40 is considered a moderate disability, and the mean score from normative data for the general population is 10.1.^36^, ^37^ Almost all patients (97.0%, 32/33) had a scapular border (±latissimus) harvest, which requires partial transection of 3 of the 4 rotator cuff muscles (teres minor, infraspinatus, subscapularis) in addition to the teres major muscle. Surprisingly, harvesting an additional chimeric latissimus muscle or performing an ipsilateral neck dissection was not associated with a change in postoperative function, which is inconsistent with prior reports.^25^ Our DASH scores were both similar or better,^23^, ^24^ or worse,^25^ than those of other studies with smaller sample sizes. Our mean DASH score was also similar to a study with a larger sample size of 120 scapular tip flaps.^38^ Although our study did not detect a change in morbidity with postoperative PT, it is possible only those patients with the worst function were referred to PT. Mobargha et al^25^ may have had better DASH scores due to standardizing shoulder PT across all postoperative patients both while inpatient and after discharge. Other studies with better postoperative scores only published scapular tip flaps,^26^, ^28^ which require a less invasive harvest technique. Unfortunately, our study did not include sufficient scapula tip patients to compare outcomes in a statistically meaningful way with those who underwent scapula border harvest. This discrepancy is likely due to the patient population in our geographic catchment area which presents with more advanced disease requiring longer segments of bone. It may also be a result of surgeons preferentially selecting the scapula flap for larger defects.

### OCRFFs

Data from our study showed that patients undergoing OCRFF reconstruction had a modest difference (13.5%) in function between the operated and nonoperated side after 1 year, with a postoperative mean (SD) DASH score of 15.6 (15.3) (Table 2). A score of 0 to 20 is considered minimal disability, and as mentioned above, the mean score from normative data for the general population is 10.1.^37^ Our mean DASH score is similar to several other studies with similar^29^ and smaller cohort sizes.^20^ In our cohort, each additional cm of the harvested radius was not associated with a change in long‐term function, which is in contrast with Arganbright et al's^39^ large study showing patients were 1.3 times more likely to have plate exposure for every increase of 1 cm of bone harvest length. Their patients also had a 35.3% donor site wound complication rate, compared with our rate of 3.6%. It is unclear why our rate is so much lower, but the only noticeable difference is they plated their radius dorsally, while we plate the volar radius. Our OCRFF patients had a fairly low rate of PT referral (39.3%), again which may be related to the overall low postoperative morbidity and therefore low desire and need to perform PT.

### Fibula Flaps

We found that patients undergoing fibula flap reconstruction had a mild difference (10.0%) in function between the operated and nonoperated side after 1 year, with a postoperative mean (SD) FAAM score of 86% (20%) (Table 3). The mean score from normative data for the general population is 92.3%.^40^ This subset of patients was least likely to be referred to postoperative PT (26.5%), and their long‐term functional status was not significantly impacted when they were referred. It is possible that this subset of patients did not need PT and therefore either were not referred or did not attend. Another possibility is that those who did attend may have only seen marginal improvements due to already adequate musculoskeletal function. Our long‐term postoperative functional scores are similar to other studies reporting scores of 89.4%,^30^ and 89%^27^ (Foot and Ankle Disability Index score: predecessor to FAAM) and Ling et al's^41^ 85.5% (American Orthopaedic Foot and Ankle Society score: not validated, clinician‐rated). We did not detect a score difference between patients with primary versus STSG closure of the donor site, which is consistent with other studies evaluating long‐term results.^42^ Patients undergoing fibula flaps had the highest rate of acute donor site wound complications (25.5%), and the presence of these complications was associated with worse long‐term functional outcomes. In other words, postoperative care of the donor site is critical to prevent and minimize acute donor site complications and potentially long‐term function in patients who undergo FFF. At our institution, we have implemented a change in wound care by adhering to orthopedic principles, uniformly requiring nonconstricting Webril dressing and splint placement for 7 days, followed by 6 weeks of walking boot usage to maximize wound healing in the acute postoperative period and potentially improve postoperative limb functional status.

### Comparison

The scapula flap has the greatest difference in long‐term musculoskeletal function when compared to OCRFF, but the overall disability is still considered on the low end of moderate. With its low donor site complication rate and versatility of soft tissue and bone, the scapula flap remains a valuable option in certain patients. The OCRFF similarly has very low donor site complication rates and an even lower difference in long‐term postoperative functional ability, which is consistent with the current literature.^21^ The fibula flap has a low difference in postoperative function between the operated and nonoperated limb and is the best substrate for dentition. However, it has the highest acute wound complication rate and greatest requirement for postoperative donor site management, a finding consistent with published reports.^22^ These complications are also associated with worse long‐term functional outcomes. Therefore, patients with more support and resources during the immediate postoperative period might be better suited for this donor site. Regardless, all patients undergoing osseous free flaps harvest would benefit from a standardized outpatient PT protocol which may mitigate morbidity and optimize functional outcomes.

### Limitations

This study has several limitations which must be addressed. First, a response rate of only 22.1% (162/731) was obtained across all patients. Furthermore, both scapula (n = 33) and OCRFF (n = 29) donor sites had low power, limiting the ability to draw robust statistical conclusions. On closer examination of the remaining 569 patients who did not respond to the survey, 83 (14.6%) were confirmed to be expired. Patients who underwent a scapula flap were most likely to have expired (27.7%, 53/191), followed by OCRFF (9.2%, 8/87), and finally fibula (4.8%, 22/454). The remaining 486 patients (66.5%) were either unreachable via telephone or refused to complete either survey. The limited survey response rate poses a self‐selection bias excluding unresponsive or expired patients and might have skewed data toward better postoperative function across all donor sites. Additionally, scapula tip harvest patients were limited in number, resulting in an inability to make meaningful conclusions about the morbidity of this specific free flap site.

Due to the cross‐sectional technique employed in our study, it was not possible to administer all surveys at the same postoperative time for each patient. An assumption was made that functional morbidity would not change significantly beyond 1 year after surgery, and this is supported by current literature.^34^, ^35^ Additionally, there are limitations regarding the use of the DASH and FAAM surveys in our study. First, both surveys are patient‐reported, resulting in a vulnerability to response bias. The DASH and FAAM were designed to monitor changes in musculoskeletal function over the course of time,^33^, ^36^ but the retrospective nature of this study did not allow for that option. The 2 surveys were not designed to be compared, so we could not make conclusions regarding the relative function between all 3 surgical sites.

## Conclusion

Donor site morbidity is a crucial factor when planning individualized reconstructive surgery. Using validated patient‐reported orthopedic surveys, patients reported a difference in long‐term function of 18.5% after a scapula border flap, 13.5% after an OCRFF, and 10.0% after an FFF. Despite the invasiveness of osseous free flap harvest, the long‐term postoperative functional morbidity is acceptable with our current techniques.

## Author Contributions

**Tyler G. Chan**, writing, data collection, analysis, revisions; **Aaron Rosado**, data collection; **Subir Goyal**, statistical analysis; **Rachel Irizarry**, revisions; **Robert J. Owen**, revisions; **Harry Michael Baddour**, revisions; **Brian Boyce**, revisions; **Azeem Kaka**, revisions; **Mark W. El‐Deiry**, revisions; **Jennifer H. Gross**, conception, writing, analysis, revisions, supervision.

## Disclosures

### Competing interests

The authors have conflicts of interest to disclose.

### Funding source

The research reported in this publication was supported in part by the Biostatistics Shared Resource of Winship Cancer Institute of Emory University and NIH/NCI under award number P30CA138292.
